# Body image concerns in patients with persecutory delusions

**DOI:** 10.1017/S0033291722000800

**Published:** 2023-07

**Authors:** Felicity Waite, Rowan Diamond, Nicola Collett, Emily Bold, Eleanor Chadwick, Daniel Freeman

**Affiliations:** 1 Department of Psychiatry, University of Oxford, Oxford, UK; 2 Oxford Health NHS Foundation Trust, Oxford, UK

**Keywords:** Appearance, delusions, obesity, paranoia, schizophrenia, weight

## Abstract

**Background:**

Persecutory fears build on feelings of vulnerability that arise from negative views of the self. Body image concerns have the potential to be a powerful driver of feelings of vulnerability. Body image concerns are likely raised in patients with psychosis given the frequent weight gain. We examined for the first-time body esteem – the self-evaluation of appearance – in relation to symptom and psychological correlates in patients with current persecutory delusions.

**Methods:**

One-hundred and fifteen patients with persecutory delusions in the context of non-affective psychosis completed assessments of body image, self-esteem, body mass index (BMI), psychiatric symptoms and well-being. Body esteem was also assessed in 200 individuals from the general population.

**Results:**

Levels of body esteem were much lower in patients with psychosis than non-clinical controls (*d* = 1.2, *p* < 0.001). In patients, body esteem was lower in women than men, and in the overweight or obese BMI categories than the normal weight range. Body image concerns were associated with higher levels of depression (*r* = *−*0.55, *p* < 0.001), negative self-beliefs (*r =* −0.52, *p* < 0.001), paranoia (*r =* −0.25, *p* = 0.006) and hallucinations (*r =* −0.21, *p* = 0.025). Body image concerns were associated with lower levels of psychological wellbeing (*r =* 0.41, *p* < 0.001), positive self-beliefs (*r =* 0.40, *p* < 0.001), quality of life (*r =* 0.23, *p* = 0.015) and overall health (*r =* 0.31, *p* = 0.001).

**Conclusions:**

Patients with current persecutory delusions have low body esteem. Body image concerns are associated with poorer physical and mental health, including more severe psychotic experiences. Improving body image for patients with psychosis is a plausible target of intervention, with the potential to result in a wide range of benefits.

## Introduction

More than half of patients with psychosis meet criteria to be classified as having obesity (Annamalai, Kosir, & Tek, 2017). Body image concerns are likely present but seldom asked about in clinical practice. Moreover, body image concerns may contribute directly and indirectly to the maintenance of psychotic experiences. Paranoia flourishes when people feel vulnerable due to feeling inferior, odd and apart (Freeman, 2016). Body image concerns likely raise the sense of vulnerability and thus directly contribute to the occurrence of paranoia. Appearance concerns may also contribute to the withdrawal that reaches agoraphobic levels in two-thirds of patients with psychosis (Freeman, Taylor, Molodynski, & Waite, 2019b) and thus indirectly contribute to a worsening of mental health. In this paper, we report for the first time the extent of body image concerns in patients with current psychotic experiences.

Concerns about body image have been shown to be associated with paranoia in the general population (Waite & Freeman, 2017). In two nationally representative datasets of adults and adolescents, totalling over 15 000 individuals, concerns about weight were significantly associated with higher levels of paranoia (Waite & Freeman, 2017). The associations remained significant after controlling for gender and body mass index (BMI). These results indicate that negative body image and paranoia are associated in the general population. Yet this association has not been tested with patients with current persecutory delusions.

Rapid weight gain in the initial phase of antipsychotic medication use is common (Pillinger et al., 2020). Qualitative research has identified that patients with psychosis often experience a negative impact from unwanted and ‘uncontrollable’ weight gain (Marshall, Freeman, & Waite, 2019; Waite et al., 2022). Patients have described how rapid weight gain, following antipsychotic medication use, compounds a loss of confidence and self-worth, and appearance concerns arise, with further consequences on mood, activity, persecutory fears, content of voices and even episodes of deliberate self-harm and suicidal ideation (Marshall et al., 2019; Waite et al., 2022). Indeed, this rapid weight gain has been described as a ‘double whammy’ of consequences, reflecting impact on both physical and mental health (Haracz, Hazelton, & James, 2018).

In patients with psychosis, excess weight is associated with low self-esteem, poor quality of life, social isolation and medication non-adherence (de Hert et al., 2006; Mccloughen & Foster, 2011). In this study, we set out to provide the first quantitative investigation of body image in patients with persecutory delusions in the context of non-affective psychosis. The primary question was whether body image concerns are likely to be prevalent in this patient population. We also tested whether body image concerns relate to the severity of psychotic experiences, and a range of other psychological, psychiatric and functioning domains.

## Method

### Participants

The participants were 115 patients with persistent persecutory delusions who had received a non-affective psychosis diagnosis. The patients were recruited as part of the Feeling Safe clinical trial for the treatment of persecutory delusions (Freeman et al., 2021) from three NHS mental health trusts: Oxford Health NHS Foundation Trust, Berkshire Healthcare NHS Foundation Trust and Northamptonshire Healthcare NHS Foundation Trust. Ethical approval was received from an NHS Research Ethics Committee (South Central – Oxford B Research Ethics Committee; reference 15/SC/0508).

The inclusion criteria for the trial were: a current, persistent (at least 3 months) persecutory delusion (as defined by Freeman & Garety, 2000), held with at least 60% conviction; a primary diagnosis of schizophrenia spectrum psychosis (non-affective psychosis); aged 16 years or above; and willing and able to give informed consent for participation in the trial. The exclusion criteria were: current receipt of another psychological therapy; insufficient comprehension of English; primary diagnosis of alcohol, drug or personality disorder; receiving treatment in a forensic service; organic syndrome; or learning disability.

Adults from the local population were recruited for a non-clinical control group. The inclusion criteria were: aged 18 years or older; ability to understand and communicate in English; and willing and able to give informed consent for participation in the study. The exclusion criterion was current receipt of help for a mental health problem or in contact with mental health services. The non-clinical group was recruited via public advertising including flyers to local postcodes and adverts on social media and local radio. A brief telephone or email screen was conducted to ensure prospective participants met the inclusion criteria. Questionnaires were completed at an assessment session at the University of Oxford. The study received ethical approval from the University of Oxford Central University Research Ethics Committee (reference R47799/RE001). Written informed consent was received from all participants.

### Measures

#### Body image

*Body-Esteem Scale for Adolescents and Adults* (*BESAA*) (Mendelson, Mendelson, & White, 2001). The BESAA is a 23-item self-report scale assessing self-evaluation of appearance. Each item (e.g. ‘I like what I look like in pictures’) is rated on a 0 (never) to 4 (always) scale. Nine items are negatively worded; these items are reverse scored. There are three subscales: ‘appearance’ includes general feelings regarding appearance (10 items) (e.g. ‘I like what I see when I look in the mirror’, ‘I feel ashamed of how I look’), ‘attribution’ includes evaluations attributed to others about one's appearance (five items) (e.g. ‘Other people consider me good looking’) and ‘weight’ relates to satisfaction with weight (eight items) (e.g. ‘I am satisfied with my weight’, ‘My weight makes me unhappy’). Higher scores indicate greater levels of positive body-esteem. In the current study, the internal consistency of the BESAA for the total sample was high for the total scale score (Cronbach's *α* = 0.94, *n* = 302) and subscale scores: appearance subscale (Cronbach's *α* = 0.91, *n* = 312), attribution subscale (Cronbach's *α* = 0.80, *n*  = 307) and weight subscale (Cronbach's *α* = 0.92, *n* = 312).

*SCOFF Questionnaire – control item* (Morgan, Reid, & Lacey, 1999). We used the single loss of control item from the questionnaire: ‘do you worry you have lost control over how much you eat?’. This item requires a dichotomous yes/no response.

*Body Mass Index* (*BMI*). This is a value derived from an individual's weight and height. It is calculated by dividing an individual's weight in kilograms by their height in meters. BMI scores can be categorised as underweight (<18.5), normal weight (18.5–24.99), overweight (25–29.99) or obese (>30). The obese range has subcategories: class I (30–34.99), class II (35–39.99) and severe (>40). To calculate BMI, participants' height and weight were measured during the assessment.

#### Psychotic experiences

*Revised Green et al. Paranoid Thoughts Scale* (*RGPTS*) (Freeman et al., 2019a). This 18-item scale measures paranoid thinking over the past month. Part A assesses ideas of reference (e.g. ‘It was hard to stop thinking about people talking about me behind my back’) and Part B assesses ideas of persecution (e.g. ‘I was convinced there was a conspiracy against me’). Each item is rated on a five-point scale. Higher scores indicate greater levels of paranoia.

*Cardiff Anomalous Perceptions Scale* (*CAPS*) (Bell, Halligan, & Ellis, 2006). Five items assessing voice hearing were used from the scale. Each item (e.g. ‘Hear voices commenting on what you're thinking or doing’, ‘Hear noise or sounds when there is nothing about to explain them’, ‘Hear two or more unexplained voices talking to each other’) is rated on a 0 (not at all) to 5 (daily) scale. Higher scores indicate greater levels of hallucinatory experiences.

*The Temporal Experience of Pleasure Scale* (*TEPS*) (Gard, Gard, Kring, & John, 2006). The 10-item anticipatory pleasure scale was used as a marker of anhedonia. Each item (e.g. ‘When I think about eating my favourite food, I can almost taste how good it is’) is rated on a six-point scale (1, very false for me to 6, very true for me) over the past month. Higher scores indicate lower levels of anticipatory pleasure.

#### Negative affect and related processes

*Brief Core Schema Scales* (*BCSS*) (Fowler et al., 2006). The BCSS comprises 24 items assessing negative and positive beliefs about the self and others over the past week. Each item is rated on a five-point scale (0–4). Four subscale scores are obtained: negative self (e.g. ‘I am unloved’), positive self (e.g. ‘I am respected’), negative other (e.g. ‘Other people are hostile’), positive other (e.g. ‘Other people are fair’). Higher scores indicate greater endorsement of items.

*Beck Depression Inventory-II* (*BDI*) (Beck, Steer, & Brown, 1996). The BDI-II is a self-report 21-item scale, with each item rated on a four-point scale (0–3), for the assessment of depression over the past fortnight. Higher scores indicate higher levels of depression.

*Columbia-Suicide Severity Rating Scale* (C-SSRS) (Posner et al., 2011). The C-SSRS is an interviewer-rated measure assessing suicidal ideation and behaviour over the past month. We have used the single severity of suicidal ideation scale in our analyses, this includes a single rating from 0 (not at all) to 5 (suicidal intent with plan). Higher scores indicate more severe suicidal ideation.

*Penn State Worry Questionnaire* (*PSWQ*) (Meyer, Miller, Metzger, & Borkovec, 1990). This 16-item scale measures trait worry over the past fortnight. Each item is rated on a five-point scale. Higher scores indicate a greater tendency to worry.

*Insomnia Severity Index* (*ISI*) (Bastien, Vallières, & Morin, 2001). The ISI is a seven-item self-report questionnaire assessing insomnia symptoms over the past fortnight. Each item is rated on a 0–4 scale. Higher scores indicate the presence of symptoms of insomnia.

*Safety Behaviours Questionnaire – Persecutory Delusions* (*SBQ*) (Freeman, Garety, & Kuipers, 2001). The SBQ is a semi-structured interview assessing safety behaviours used in the last month. An action is deemed a safety behaviour if the interviewee reports that it has been carried out with the intention of reducing persecutory threat. A distinction is made between avoidance of situations and in-situation safety behaviours (e.g. not making eye contact). The number and frequency of safety behaviours are calculated to produce a total score. Frequency is rated on a four-point scale from 1 (behaviour definitely occurred on at least one occasion) to 4 (present more or less continuously/at least every day). Higher scores indicate a greater number and frequency of safety behaviours.

#### Quality of life

*Warwick-Edinburgh Mental Well-being Scale* (*WEMWBS*) (Tennant et al., 2007). The WEMWBS is a 14-item scale assessing well-being over the past fortnight. Each item is rated on a 1 (none of the time) to 5 (all of the time) scale, and therefore the total score can range from 14 to 70, with higher scores indicating a greater level of well-being.

*EuroQoL* (*EQ-5D-5*) (Herdman et al., 2011). We used two scores from the questionnaire: quality of life and overall health. Quality of life is reported using the EQ-5D crosswalk index value. This is a dimensional score between 0 and 1 calculated using EuroQol index data for five items each scored 1–5. Higher scores indicate better quality of life. For overall health, a rating scale (0–100) is used to indicate ‘how good is your health today’. Higher scores indicate better overall health.

*Time budget* (*TB*) (Jolley et al., 2006). The time budget, completed during a structured interview, assesses meaningful activity levels over the past week, with four time-blocks for each day rated from 0 to 4. The rating scale is: 0 = nothing, 1 = predominantly passive activity, 2 = an independent activity requiring some planning and motivation, 3 = several two-rated activities completely filling a time period or a more complex and demanding, but shorter, activity, 4 = time period filled with a variety of demanding independent activities. Higher scores indicate higher levels of meaningful activity.

### Analyses

To test group differences on the body image measure, *t* tests were employed. A Cohen's *d* calculation was used to determine effect sizes. Associations between body image and the symptom and psychological variables within the clinical group were tested using Pearson correlations. To test differences between gender and BMI categories, *t* tests, χ^2^ tests and ANOVAs were conducted. All statistical testing was two-tailed and carried out with SPSS Version 27.0 (IBM, 2020).

## Results

### Demographic and clinical information

#### Clinical group

The average age of the patients was 41.8 years (s.d. = 11.8; range 17–62). There were slightly more men (*n* = 69, 60%) than women (*n* = 46, 40%). The ethnicities were: White (*n* = 98, 85.2%), Black Caribbean (*n* = 7, 6.1%), Indian (*n* = 3, 2.6%), Pakistani (*n* = 2, 2.6%), Black African (*n* = 2, 1.7%), Chinese (*n* = 1, 0.9%) and other (*n* = 1, 0.9%). Most participants in the clinical group were single (*n* = 81, 70.4%), with others married or in a civil partnership (*n* = 22, 19.1%), co-habiting (*n* = 2, 1.7%) or divorced (*n* = 10, 8.7%). The majority were unemployed (*n* = 91, 79%). Three-quarters (74.1%) of patients had a BMI in the overweight (*n* = 31, 27.7%) or obese (*n* = 52, 46.4%) range. No patients had a BMI in the underweight range. The clinical diagnoses were schizophrenia (*n* = 71, 61.7%), schizoaffective disorder (*n* = 21, 18.3%), delusional disorder (*n* = 3, 2.6%) and psychosis NOS (*n* = 20, 17.4%). All but four of the patients (*n* = 111, 96.5%) were currently prescribed anti-psychotic medication. The mean antipsychotic defined daily dose was 1.50 (s.d. = 0.79) and the mean chlorpromazine equivalent dose was 473.97 (s.d. = 396.04). Almost all participants were outpatients (*n* = 111, 96.5%) at the time of participation. Table 1 reports the mean scores for all clinical measures.

**Table 1.** Mean scores on the measures in the clinical group

Variable	*n*	Mean	s.d.
Body esteem (BESAA)	115	30.18	16.2
Body esteem – appearance subscale	112	15.41	8.28
Body esteem – attribution subscale	107	5.50	3.87
Body esteem – weight subscale	112	9.67	6.71
Body mass index	112	30.23	6.94
Paranoia –ideas of reference (RGPTS- part A)	115	17.29	7.75
Paranoia – persecutory ideation (RGPTS- part B)	115	26.97	8.60
Hallucinations (CAPS)	115	23.11	14.64
Anhedonia (TEPS)	115	29.80	11.60
Negative-self beliefs (BCSS)	115	12.06	5.52
Positive-self beliefs (BCSS)	115	7.47	4.99
Depression (BDI)	115	31.48	12.09
Suicidal ideation (CSSRS)	115	1.97	1.56
Worry (PSWQ)	115	63.04	10.60
Insomnia (ISI)	115	14.23	6.74
Safety-seeking behaviours (SBQ)	114	35.38	16.81
Wellbeing (WEMWBS)	115	34.16	8.17
Quality of life (EQ5D)	115	0.51	0.25
Overall health (EQ5D)	115	48.60	20.51
Activity (time budget)	115	53.38	14.96

#### Non-clinical control group

The general population group comprised 100 men and 100 women. The mean age was 39.2 years (s.d. = 13.4; range 18–76). The mean BMI was 26.7 (s.d. = 5.80, range 15–60.5). The majority of participants (58.5%) had a BMI in the overweight (*n* = 72, 36%) or obese (*n* = 45, 23%) range. Seventy-eight participants (39%) had a BMI in the healthy weight range, and only four participants (2%) had a BMI in the underweight range. There was missing BMI data for one participant. The reported ethnicities were: White (*n* = 186, 93%), Asian (*n* = 7, 3.5%), Black African (*n* = 1, 0.5%) and other (*n* = 6, 3.0%). The majority of participants (*n* = 166, 83%) were working either full-time (*n* = 99, 49.5%), part-time (*n* = 36, 18%), or self-employed (*n* = 31, 15.5%). With others in full-time education (*n* = 18, 9%), unemployed (*n* = 9, 4.5%), retired (*n* = 5, 2.5%) or a househusband/housewife (*n* = 2, 1%). Most participants in the general population group were married or in a civil partnership (*n* = 78, 39%), single (*n* = 64, 32%) or co-habiting (*n* = 38, 19%), with others divorced or widowed (*n* = 20, 10%).

There was no statistically significant difference in the mean age of the two groups: [*t*(312) = −1.73, *p* = 0.84]. There was a non-significant higher proportion of men in the clinical group (*n* = 69, 60%) than the non-clinical group (*n* = 100, 50%), χ(1) = 2.936, *p* = 0.087. The clinical group had a higher mean BMI [*t*(309) = 4.86, *p* < 0.001], with a mean difference of 3.58 (CI 2.13–5.03). There was a significant difference between the groups for employment status, with significantly fewer participants in the clinical group in employment [χ(1) = 149.561, *p* < 0.001].

### Body image in patients with psychosis

Table 2 summarises the group scores on the body esteem scale and subscales. In comparison to the control group, levels of body esteem were low in patients with psychosis. Differences were seen on all three subscales, with patients reporting lower body esteem in relation to appearance, weight and attribution than participants in the non-clinical control group. There were large effect size differences in total score, and on the appearance and attribution subscales, with a medium effect size on the weight subscale. These differences remained, though with slightly reduced effect sizes, when comparing a subsample of participants with a BMI in the overweight category from the patient group (*n* = 52) and non-clinical group (*n* = 117), for the total body esteem score [*t*(167) = −7.723, *p* < 0.001, Cohen's *d* = 0.66, CI −1.640 to −0.931], and the appearance [*t*(165) = −6.400, *p* < 0.001, Cohen's *d* = 0.77, CI −1.431 to −0.729], weight [*t*(165) = −5.574, *p* < 0.001, Cohen's *d* = 0.74, CI −1.87 to −0.594] and attribution [*t*(163) = −6.630, *p* < 0.001, Cohen's *d* = 0.71, CI −1.493 to −0.777] subscales.

**Table 2.** Comparison of body esteem in clinical and non-clinical samples: means, standard deviations, *t* values, confidence intervals, *p* values and effect sizes for body esteem

Measure	*n*		Mean (s.d.)		*t* Value (df)	95% CI	*p* Value	Effect size	
	Clinical	Non-clinical	Clinical	Non-clinical				Cohen's *d*	95% CI
Body esteem – total	115	200	1.31 (0.70)	2.14 (0.68)	−10.32 (313)	−0.99 to −0.67	*p* < 0.001	−1.21	−1.46 to −0.96
Body esteem – appearance subscale	112	200	1.54 (0.83)	2.39 (1.54)	−9.42 (310)	−1.02 to −0.67	*p* < 0.001	−1.11	−1.36 to −0.86
Body esteem – weight subscale	112	200	1.21 (0.84)	1.99(1.21)	−6.05 (310)	−0.79 to −0.40	*p* < 0.001	−0.71	−0.95 to −0.48
Body esteem – attribution subscale	107	200	1.10 (0.77)	1.90 (1.10)	−9.52 (305)	−0.97 to −0.64	*p* < 0.001	−1.14	−1.39 to −0.89

The frequency of endorsement for each scale item, for both the patient and non-clinical participants, is shown in Table 3. The patterns of endorsement indicate clear differences between the two groups, for example, on item 17, ‘*I feel ashamed of how I look*’ around 80% of participants in the non-clinical group endorse this item never or seldom, whereas the reverse pattern is seen in the patient group, with most (around 70%) endorsing this item sometimes, often or always. There are also distinct differences in particular items which indicate the impact of body image concerns, for example, item 13 ‘*My looks upset me*’ highlights the emotional toll of body image concerns for patients. Three-quarters of participants in the non-clinical group endorse this item never or seldom. In contrast, three-quarters of participants in the clinical group endorse this item sometimes, often or always.

**Table 3.** Item endorsement (*n*, %) on the BESAA

BESAA item	*n*	Clinical					*n*	Non-clinical				
		Never	Seldom	Sometimes	Often	Always		Never	Seldom	Sometimes	Often	Always
1.	I like what I look like in pictures.	115	38 (33.0%)	35 (30.4%)	32 (27.8%)	7 (6.1%)	3 (2.6%)	200	7 (3.5%)	57 (28.5%)	100 (50.0%)	35 (17.5%)	1 (0.5%)
2.	Other people consider me good looking.	114	29 (25.2%)	42 (36.5%)	31 (27.0%)	10 (8.7%)	2 (1.7%)	200	6 (3.0%)	30 (15.0%)	113 (56.5%)	47 (23.5%)	4 (2.0%)
3.	I'm proud of my body.	112	53 (46.1%)	35 (30.4%)	18 (15.7%)	4 (3.5%)	2 (1.7%)	198	23 (11.5%)	62 (31.0%)	76 (38.0%)	31 (15.5%)	6 (3.0%)
4.	*I am preoccupied with trying to change my body weight.	115	16 (13.9%)	21 (18.3%)	32 (27.8%)	26 (22.6%)	20 (17.4%)	200	32 (16.0%)	50 (25.0%)	50 (25.0%)	43 (21.5%)	25 (12.5%)
5.	I think my appearance would help me get a job.	115	47 (40.9%)	35 (30.4%)	21 (18.3%)	8 (7.0%)	4 (3.5%)	199	55 (27.5%)	50 (25.0%)	56 (28.0%)	26 (13.0%)	12 (6.0%)
6.	I like what I see when I look in the mirror.	115	42 (36.5%)	33 (28.7%)	31 (27.0%)	7 (6.1%)	2 (1.7%)	200	11 (5.5%)	34 (17.0%)	116 (58.0%)	36 (18.0%)	3 (1.5%)
7.	*There are lots of things I'd change about my looks if I could.	115	9 (7.8%)	10 (8.7%)	37 (32.2%)	33 (28.7%)	26 (22.6%)	200	24 (12.0%)	62 (31.0%)	55 (27.5%)	42 (21.0%)	17 (8.5%)
8.	I am satisfied with my weight.	115	57 (49.6%)	28 (24.3%)	19 (16.5%)	9 (7.8%)	2 (1.7%)	200	42 (21.0%)	50 (25.0%)	54 (27.0%)	37 (18.5%)	17 (8.5%)
9.	*I wish I looked better.	115	7 (6.1%)	6 (5.2%)	32 (27.8%)	30 (26.1%)	40 (34.8%)	200	12 (6.0%)	34 (17.0%)	77 (38.5%)	52 (26.0%)	25 (12.5%)
10.	I really like what I weigh.	115	58 (50.4%)	26 (22.6%)	24 (20.9%)	7 (6.1%)	0 (0.0%)	200	53 (26.5%)	64 (32.0%)	40 (20.0%)	31 (15.5%)	12 (6.0%)
11.	*I wish I looked like someone else.	114	37 (32.3%)	18 (15.7%)	31 (27.0%)	11 (9.6%)	17 (14.8%)	200	98 (49.0%)	46 (23.0%)	41 (20.5%)	11 (5.5%)	4 (2.0%)
12.	People my own age like my looks.	115	34 (29.6%)	38 (33.0%)	30 (26.1%)	9 (7.8%)	4 (3.5%)	200	8 (4.0%)	24 (12.0%)	97 (48.5%)	67 (33.5%)	4 (2.0%)
13.	*My looks upset me.	115	18 (15.7%)	14 (12.2%)	44 (38.3%)	20 (17.4%)	19 (16.5%)	200	92 (46.0%)	57 (28.5%)	41 (20.5%)	6 (3.0%)	4 (2.0%)
14.	I'm as nice looking as most people.	112	27 (23.5%)	34 (29.6%)	35 (30.4%)	10 (8.7%)	6 (5.2%)	200	5 (2.5%)	33 (16.5%)	74 (37.0%)	61 (30.5%)	27 (13.5%)
15.	I'm pretty happy about the way I look.	114	29 (25.2%)	32 (27.8%)	39 (33.9%)	11 (9.6%)	2 (1.7%)	200	4 (2.0%)	34 (17.0%)	65 (32.5%)	78 (39.0%)	19 (9.5%)
16.	I feel I weigh the right amount for my height.	115	60 (52.2%)	22 (19.1%)	20 (17.4%)	10 (8.7%)	3 (2.6%)	200	43 (21.5%)	53 (26.5%)	36 (18.0%)	43 (21.5%)	25 (12.5%)
17.	*I feel ashamed of how I look.	115	10 (8.7%)	28 (24.3%)	30 (26.1%)	25 (21.7%)	22 (19.1%)	200	115 (57.5%)	44 (22.0%)	30 (15.0%)	9 (4.5%)	2 (1.0%)
18.	*Weighing myself depresses me.	115	22 (19.1%)	16 (13.6%)	26 (22.6%)	26 (22.6%)	25 (21.7%)	200	88 (44.0%)	40 (20.0%)	34 (17.0%)	21 (10.5%)	17 (8.5%)
19.	*My weight makes me unhappy	115	14 (12.2%)	19 (16.5%)	30 (26.1%)	24 (20.9%)	28 (24.3%)	200	75 (37.5%)	50 (25.0%)	38 (19.0%)	21 (10.5%)	16 (8.0%)
20.	My looks help me to get dates.	111	69 (60.0%)	26 (22.6%)	14 (12.2%)	1 (0.9%)	1 (0.9%)	199	53 (26.5%)	42 (21.0%)	70 (35.0%)	30 (15.0%)	4 (2.0%)
21.	*I worry about the way I look.	114	13 (11.3%)	14 (12.2%)	34 (29.6%)	26 (22.6%)	27 (23.5%)	200	49 (24.5%)	53 (26.5%)	56 (28.0%)	26 (13.0%)	15 (7.5%)
22.	I think I have a good body.	115	60 (52.2%)	31 (27.0%)	17 (14.8%)	6 (5.2%)	1 (0.9%)	200	33 (16.5%)	48 (24.0%)	74 (37.0%)	40 (20.0%)	5 (2.5%)
23.	I'm looking as nice as I'd like to.	115	40 (34.8%)	39 (33.9%)	25 (21.7%)	5 (4.3%)	6 (5.2%)	200	26 (13.0%)	53 (26.5%)	72 (36.0%)	43 (21.5%)	6 (3.0%)

*Negative items, which must be recoded for scoring by reversing the scale (i.e. 0 = 4, 1 = 3, 2 = 2, 3 = 1, 4 = 0). Three subscales: appearance (1, 6, 7*, 9*, 11*, 13*, 15, 17*, 21*, 23); weight (3, 4*, 8, 10, 16, 18*, 19*, 22); and attribution (2, 5, 12, 14, 20).

In comparison to other published studies using the BESAA, the patients with psychosis scored lower on the total and subscale means in contrast to: studies with student (Mendelson, Mendelson, & Andrews, 2000), middle-aged (McLaren & Kuh, 2004) and young adolescent (Cragun, DeBate, Ata, & Thompson, 2013) participants, studies comparing sex differences in body esteem (Brennan, Lalonde, & Bain, 2010), and studies conducted in different countries [e.g. Sweden (Ivarsson, Svalander, Litlere, & Nevonen, 2006) or Korea (Jun & Choi, 2014)]. A table of comparative studies to allow benchmarking is provided in the online Supplementary materials (online Supplementary Table S1).

#### Gender

In the patient group, there were significant differences between male (*M* = 34.25, s.d. = 15.73) and female (*M* = 24.08, s.d. = 15.11) participants in total body esteem [*t*(113) = 3.45, *p* = 0.001] and for the appearance [*t*(110) = 3.37, *p* = 0.001] and weight [*t*(110) = 4.04, *p* < 0.001] subscales, but not the attribution subscale [*t*(105) = 0.45, *p* = 0.66]. In contrast, there were no significant differences between male (*M* = 50.45, s.d. = 14.66) and female (*M* = 48.05, s.d. = 16.35) participants in total body esteem [*t*(198) = 1.094, *p* = 0.275] or on the appearance [*t*(198) = 1.272, *p* = 0.205], weight [*t*(198) = 1.906, *p* = 0.058] and attribution [*t*(198) = −1.543, *p* = 0.275] subscales in the non-clinical group. This is distinct from previous findings using the BESAA in which gender differences are commonly observed (Cragun et al., 2013).

#### BMI categories

There were significant differences between BMI categories in the level of body esteem in both the clinical [*F*(_2,109_) = 8.199, *p* < 0.001] and non-clinical [*F*_(3,195)_ = 7.407, *p* < 0.001] groups. A Tukey post hoc test revealed that in the clinical group, levels of body esteem were statistically significantly lower in the overweight (1.29 ± 0.69, *p* = 0.022) and obese (1.13 ± 0.64, *p* < 0.001) BMI groups than the normal weight (1.74 ± 0.65) category. There was no statistically significant difference between the overweight and obese BMI category groups (*p* = 0.552). Similarly, in the non-clinical group, a Tukey post hoc test revealed that levels of body esteem were statistically significantly lower in the overweight (2.06 ± 0.72, *p* = 0.010) and obese (1.86 ± 0.57, *p* < 0.001) BMI groups than the normal weight (2.40 ± 0.57) category. There were no statistically significant differences between the underweight BMI category with any other category, nor between the overweight and obese BMI category groups (*p* = 0.345). Table 4 reports the total mean scores on the BESAA by BMI category (including obese subcategories) for the clinical group.

**Table 4.** Mean scores on the BESAA by BMI category in the clinical group

BMI category	Variable	*n*	Mean	s.d.
Normal weight	BESAA – total mean	29	1.74	0.65
	Appearance subscale	28	1.80	0.74
	Attribution subscale	27	1.38	0.86
	Weight subscale	28	1.92	0.86
Overweight	BESAA – total mean	31	1.29	0.68
	Appearance subscale	31	1.46	0.82
	Attribution subscale	29	1.01	0.62
	Weight subscale	31	1.25	0.78
Obese	BESAA – total mean	52	1.31	0.64
	Appearance subscale	50	1.50	0.85
	Attribution subscale	48	1.03	0.78
	Weight subscale	50	0.84	0.58
Obese – category I	BESAA – total mean	28	1.36	0.62
	Appearance subscale	27	1.80	0.74
	Attribution subscale	26	1.14	0.83
	Weight subscale	27	1.06	0.55
Obese – category II	BESAA – total mean	16	0.78	0.52
	Appearance subscale	16	1.04	0.78
	Attribution subscale	14	0.69	0.57
	Weight subscale	16	0.59	0.53
Obese – severe	BESAA – total mean	8	1.04	0.70
	Appearance subscale	7	1.39	1.02
	Attribution subscale	8	1.28	0.85
	Weight subscale	7	0.57	0.46

#### Eating habits

Fifty-eight (51.8%) patients reported a loss of control over eating. There were significant differences in body esteem [*t*(110) = 4.30, *p* < 0.001] between those reporting a loss of control over eating (*M*  = 1.06, s.d. = 0.66) and those who did not (*M* = 1.59, s.d. = 0.64). Loss of control was more commonly reported by patients with a BMI in the obese category than the normal weight category: χ(1) = 5.567, *p* = 0.018. However, there was no significant difference in loss of control over eating between the overweight and obese [χ(1) = 1.791, *p* = 0.181] or normal and overweight [χ(1) = 0.907, *p* = 0.341] BMI categories.

### Correlates of body esteem

Table 5 reports the correlations between body esteem and the symptom variables and psychological constructs.

**Table 5.** Correlates of body image in patients with persecutory delusions

Variable	*n*		
Paranoia – ideas of reference (RGPTS – part A)	115	−0.243**	*p* = 0.009
Paranoia – persecutory ideation (RGPTS – part B)	115	−0.254**	*p* = 0.006
Hallucinations (CAPS)	114	−0.210*	*p* = 0.025
Anhedonia (TEPS)	114	−0.335***	*p* < 0.001
Negative-self beliefs (BCSS)	115	−0.516***	*p* < 0.001
Positive-self beliefs (BCSS)	115	0.397***	*p* < 0.001
Depression (BDI)	115	−0.553***	*p* < 0.001
Suicidal ideation (CSSRS)	115	−0.321**	*p* < 0.001
Worry (PSWQ)	115	−0.437***	p < 0.001
Insomnia (ISI)	115	−0.300**	*p* = 0.001
Safety-seeking behaviours (SBQ)	114	−0.384***	*p* < 0.001
Wellbeing (WEMWBS)	115	0.414***	*p* < 0.001
Quality of life (EQ5D)	115	0.226*	*p* = 0.015
Overall health (EQ5D)	115	0.314**	*p* = 0.001
Activity (Time budget)	113	−0.033	*p* = 0.728

*p < 0.05, **p < 0.01, ***p < 0.001.

Body esteem had large negative associations with depression and negative self-beliefs (low self-esteem). There were medium effect size negative correlations between body esteem and worry, safety-seeking behaviours, anhedonia, suicidal ideation and insomnia. There were significant positive correlations of a medium effect size between body esteem and wellbeing, positive beliefs about the self (self-esteem) and overall health status. There were small effect size associations between body esteem and psychotic experiences: ideas of reference, persecutory ideation and hallucinations. All associations with psychotic experiences remained significant after controlling for antipsychotic medication dose (defined daily dose and chlorpromazine equivalent). No correlation (*r* *<* 0.1) was found between body esteem and meaningful activity as measured on the time budget. However, a small effect size association was found with quality of life as measured on the EQ5D.

## Discussion

Body image concerns are common in patients experiencing current persecutory delusions. They are associated with depression, negative self-views, suicidal ideation, worry, anhedonia, insomnia and excess weight. As might be expected, body image concerns were most strongly associated with depression and negative self-concept. Of particular interest are the significant associations with psychotic experiences: we found that body image concerns were associated with higher levels of paranoia and voices. This fits with our view that body image concerns may feed into the vulnerability underlying paranoia and into the content of voices. Body image concerns may be a meaningful treatment target in patients with persecutory delusions.

Levels of body esteem were low in patients with psychosis compared to data reported in studies from the general population with participants across a range of age, ethnicity and locations (Brennan et al., 2010; Cragun et al., 2013; Ivarsson et al., 2006; Jun & Choi, 2014; McLaren & Kuh, 2004; Mendelson et al., 2000).

In our study, there were clear gender differences in the clinical group, with women reporting lower body esteem. This is a common pattern in studies of body image, yet it may reflect a measurement issue. The items on the body esteem scale do not include muscular strength or build, features often considered important in male body image (Cragun et al., 2013). Body esteem was lower in those with a BMI in the overweight or obese categories. It was notable in the current study that body image concerns were higher in patients with psychosis even compared to non-clinical individuals in the same BMI categories. Given the elevated rates of obesity in patients with psychosis (Annamalai et al., 2017), tackling excess weight may be important for both physical and mental health (Firth et al., 2019). Indeed, body image concerns differ between patients with and without metabolic syndrome (de Hert et al., 2006). Yet the potential contribution of poor physical health to mental health problems has, to date, been overlooked. The patterns of endorsement on the individual scale items highlight differences between patients and the non-clinical control group in specific cognitions about appearance as well as the emotional impact of appearance concerns (e.g. ‘*I feel ashamed of how I look*’, ‘*My looks upset me*’). These cognitions and emotional responses could be treatment targets within a cognitive therapy intervention.

In this study, we found that in patients with psychosis, as seen in the general population (Waite & Freeman, 2017), body image concerns were associated with paranoia. Consistent with qualitative studies of patient accounts (Marshall et al., 2019; Waite et al., 2022), we found that body image concerns were also associated with negative self-concept, suicidal ideation and hearing voices. Preliminary research indicates that body image concerns are a common feature in the content of voices (Waite et al., 2019). In a study with 60 patients with psychosis, 90% reported hearing voices comment negatively on their appearance, and 50% heard negative comments about appearance on a daily basis. The most commonly endorsed item was ‘the voices tell me that I am ugly’. Whilst concerns about appearance might lead to additional safety-seeking or defence behaviours, also reported in the general population (McLaren & Kuh, 2004), in the current study, the relationship with functioning was less clear. These findings indicate multiple routes through which appearance concerns may contribute to the occurrence of psychotic experiences. For example, within the threat-anticipation model of persecutory beliefs (Freeman, 2016), concerns about appearance may be a feature of negative self-concept, a source of worry or a cause for employing safety-seeking or defence behaviours, each of which contribute to the occurrence of threat beliefs. As body image concerns are associated with psychotic experiences, as well as putative psychological causal mechanisms including low self-esteem, depression and worry (Freeman, 2016), it may provide a novel target for treatment in patients with psychosis.

### Limitations

There are a number of limitations to the current study. Firstly, it is unknown how representative the current patient participants are of the wider population of patients with non-affective psychosis. The non-clinical control group was recruited via local advertising and will not have been representative of the general population, nor was this group matched to the patient group. Secondly, in this exploratory study, with many conceptually-related variables, there was no correction for multiple testing, which raises the likelihood of Type 1 errors (false positives). Thirdly, measures of body image are commonly developed and validated with students, with high proportions of female respondents under the age of 25 years. This may result in a degree of measurement error, for example, potentially underestimating body image concerns in male participants. Reviews of body image measures, both self-report and those using other methods such as figural drawings, highlight limitations in cultural sensitivity and use across different populations (Gardner & Brown, 2010; Kling et al., 2019). This is particularly important given the intersection of body image with gender, culture, sexuality and other factors. The only questionnaire validated in this patient group (Awad & Voruganti, 2004) focuses on body weight rather than the multidimensional construct of body image (Arbour-Nicitopoulos, Faulkner, & Cohn, 2010). Although BMI was reported, other measures of physical health such as metabolic parameters or waist circumference were not included in this study. Finally, given the cross-sectional design of the study, it is not possible to determine directions of effect or the potential impact of confounding variables. Interventionist-causal designs (Kendler & Campbell, 2009) are now required to determine the effect of improving body image on mental and physical health.

## Supplementary material

For supplementary material accompanying this paper visit https://doi.org/10.1017/S0033291722000800. click here to view supplementary material
