# Supplementary material for: Body image concerns in patients with persecutory delusions
Source: Psychol Med. 2022 Apr 7;53(9):4121–9. doi: 10.1017/S0033291722000800 (PMC10317811; doi:10.1017/S0033291722000800)
Supplement: Supplementary file 1 [file S0033291722000800sup001.docx]

Supplementary table 1. BESAA scores across different samples.

| Reference |  | Sex | Age | | BMI | Total Score | | Appearance subscale | | Weight subscale | | Attribution subscale | | |
| --- | --- | --- | --- | --- | --- | --- | --- | --- | --- | --- | --- | --- | --- | --- |
|  | *n* | *n* | *M* | *SD* | *M, SD* | *M* | *SD* | *M* | *SD* | *M* | *SD* | *M* | *SD* |  |
| Waite, F. et al., clinical group | 115 | F:46 (40%)  M: 69 (60%) | 41.8 | 11.8 | M = 30.23  SD =6.94  27% overweight  44% obese | 1.31 | 0.70 | 1.54 | 0.83 | 1.21 | 0.84 | 1.10 | 0.77 |  |
| Waite, F. et al., non-clinical control group | 200 | F: 100 (50%)  M: 100 (50%) | 39.2 | 13.4 | M = 26.7  36% overweight  23% obese | 2.14 | 0.68 | 2.39 | 1.54 | 1.99 | 1.21 | 1.90 | 1.10 |  |
| Mendelson, M.J., Mendelson, B.K.., & Andrews, J. (2000). | 217 | F: 110  M: 107 | 18.2 | 1.0 | Not reported | Not reported. |  | 2.5 | 0.8 | 2.4 | 1.0 | 2.3 | 0.7 |  |
| McLaren, L., & Kuh, D. (2004) | 1026 | F:1026 (100%)  M: 0 | 54 | Not reported | M = 26.6  SD= 4.9  0.6% underweight  41.7% normal weight  36.6% overweight  21.2% obese. | No total score - attribution scale not included. |  | 3.3 | 0.7 | 2.9 | 0.9 | Not used. | N/A |  |
| Green, S., & Pritchard, M. (2003). | 139 | F: 94  (67.6%)  M: 45 (32.4%) | 42 | 11.65 | Not reported. | F: 2.86*  M: 3.62* | F: 0.72  M:0.71 | Not reported – only 14 items used from scale | | | | | |  |
|  |  |  |  |  |  |  |  |  |  |  |  |  |  |  |
| Cragun, D., DeBate, R.D., Ata, R. N., &Thompson JK. (2018). | 299 | F: 151  (51.2%)  M: 146  (48.8%) | 11.9 | 0.54 | 3 % underweight  61.5% normal weight  17.1 % at risk for overweight  18.4% overweight | No total score - attribution subscale not included. | N/A | F:  3.70  M: =3.76 | F:  0.94  M:  0.86 | F:  3.70  M: 3.89 | F:  1.07  M: 0.89 | Not used | N/A |  |
| Brennan, M.A., Lalonde, C.E., & Bain, J.L. (2010). | 197 | F: 97  M: 98 | 19.30 | 3.14 | Not reported. | Not reported. |  | F: 2.33  M: 2.60 | F: 0.69  M: 0.75 | F: 2.31  M: 2.65 | F: 0.83  M: 0.86 | F: 2.31  M: 2.41 | F: 0.68  M: 0.57 |  |
| Ivarsson, T., Svalander, P., Litlere, O., & Nevonen, L. (2006). | 405 | F: 210  (52.2%)  M: 192  (47.8%) | F: 14.7  M: 15.6 | Not reported. | F: 19.6  (SD=2.50)  M: 20.4 (SD=2.74) | F: 55.0  M: 63.5 | F: 17.03  M: 14.82 | F: 25.4  M: 28.8 | F: 7.98  M: 6.99 | F: 18.3  M: 23.4 | F: 8.43 M: 6.46 | F: 11.1  M: 11.3 | F: 3.60M: 3.93 |  |
| Jun, E., & Choi, S. (2014). | 700 | F: 700 (100%)  M: 0 | M = 20.2 | SD = 1.9 | M = 20.33 kg/m2  Underweight = 15.7%  Normal weight = 80.6%  Obese = 3.8% | 29.3**  *Mean item score: 2.44* | 4.62 | Not reported – only 12 items used from scale | | | | | |  |
| Mendelson, B.K., McLaren, L., Gauvin, L., & Steiger, H. (2002). | 177  74 ED***,  103 control) | F: 177 (100%)  M: 0 | ED: 28.09  NC: 21.96 | ED: 9.15  NC: 5.02 | ED: M 1967,  SD =4.74  NC: M 21.46,  SD = 4.00 |  |  | ED: 0.97  NC: 2.58 | 0.65  0.71 | ED: 0.82  NC:  2.69 | 0.66  0.71 | ED: 1.86  NC:  2.24 | 0.79  0.66 |  |

**only used 14 questions from measure; **only used 12 questions from measure; ** *Eating disorder diagnoses: 25% restricting type (AN); 75% binge purge type BN/AN/BED/ED-NOS*
